# Features and Possible Applications of Plant Lipid-Binding and Transfer Proteins

**DOI:** 10.3390/membranes13010002

**Published:** 2022-12-20

**Authors:** Daria N. Melnikova, Ekaterina I. Finkina, Ivan V. Bogdanov, Andrey A. Tagaev, Tatiana V. Ovchinnikova

**Affiliations:** 1 M.M. Shemyakin & Yu.A. Ovchinnikov Institute of Bioorganic Chemistry, the Russian Academy of Sciences, Miklukho-Maklaya Str., 16/10, 117997 Moscow, Russia; 2 Phystech School of Biological and Medical Physics, Moscow Institute of Physics and Technology (State University), 141701 Dolgoprudny, Russia

**Keywords:** lipid-binding and transfer protein, pathogenesis-related class 10 proteins, acyl-CoA-binding protein, puroindoline, lipid ligand, lipid binding

## Abstract

In plants, lipid trafficking within and inside the cell is carried out by lipid-binding and transfer proteins. Ligands for these proteins are building and signaling lipid molecules, secondary metabolites with different biological activities due to which they perform diverse functions in plants. Many different classes of such lipid-binding and transfer proteins have been found, but the most common and represented in plants are lipid transfer proteins (LTPs), pathogenesis-related class 10 (PR-10) proteins, acyl-CoA-binding proteins (ACBPs), and puroindolines (PINs). A low degree of amino acid sequence homology but similar spatial structures containing an internal hydrophobic cavity are common features of these classes of proteins. In this review, we summarize the latest known data on the features of these protein classes with particular focus on their ability to bind and transfer lipid ligands. We analyzed the structural features of these proteins, the diversity of their possible ligands, the key amino acids participating in ligand binding, the currently known mechanisms of ligand binding and transferring, as well as prospects for possible application.

## 1. Introduction

Lipids are essential components of many biological processes in all living organisms and serve as the building blocks of biological membranes or specific proteins, substrates for metabolic energy production, and signaling compounds. As lipophilic molecules cannot move freely in an aqueous cellular environment, several modes of transport exist. These include membrane contact sites, diffusion and/or flip transfer within the same membrane system, vesicular trafficking, and protein-mediated transport processes [1]. Many proteins are involved in delivery to, flip transfer across, and movement out of membranes [2]. For example, ABC transporters [3] and PIN [4] have been described to transport lipid molecules across various membranes; lipid transfer proteins (LTPs) [5] and pathogenesis-related class 10 (PR-10) proteins [6] facilitate non-vesicular lipid transfer to and out of membranes. One of the important features of lipid transport is the mechanism by which proteins carry it out. The study of the mechanisms of lipid binding and transport is often a laborious and complex task. A rather limited amount of pioneering work is devoted to the study of how protein–lipid complexes are formed and how ligands are transported and released at their destination. Therefore, to date, the mechanisms of lipid binding and transport are unknown for many classes of lipid-binding proteins. In this review, we considered plant lipid-binding proteins, which perform transport of various hydrophobic molecules inside the cell, in the apoplast, intercellular space, and via phloem and xylem vessels. Such proteins bind and transfer building and signaling lipid molecules, secondary metabolites with different biological activity due to which they play an important role in the growth and development of plants as well as in their protection under abiotic and biotic stress conditions. Many similar proteins have been found in different plants, including such classes as acyl carrier proteins (ACPs) [7], glycolipid transfer proteins (GLTPs) [8], etc. However, the most common and represented in plants are four classes of proteins, namely: lipid transfer proteins (LTPs), pathogenesis-related class 10 (PR-10) proteins, acyl-CoA-binding proteins (ACBPs), and puroindolines (PINs). Here, we summarize the latest data on the features of the functioning of these different, but at the same time similar, proteins, focusing on their ability to bind and transfer lipid ligands. We analyzed the structural features of these four classes of proteins, the diversity of their possible ligands, the key amino acids participating in ligand binding, the currently known mechanisms of ligand binding and transferring, as well as prospects for possible application. 

## 2. Features of Plant Lipid-Binding and Transfer Proteins

**Figure 1:**
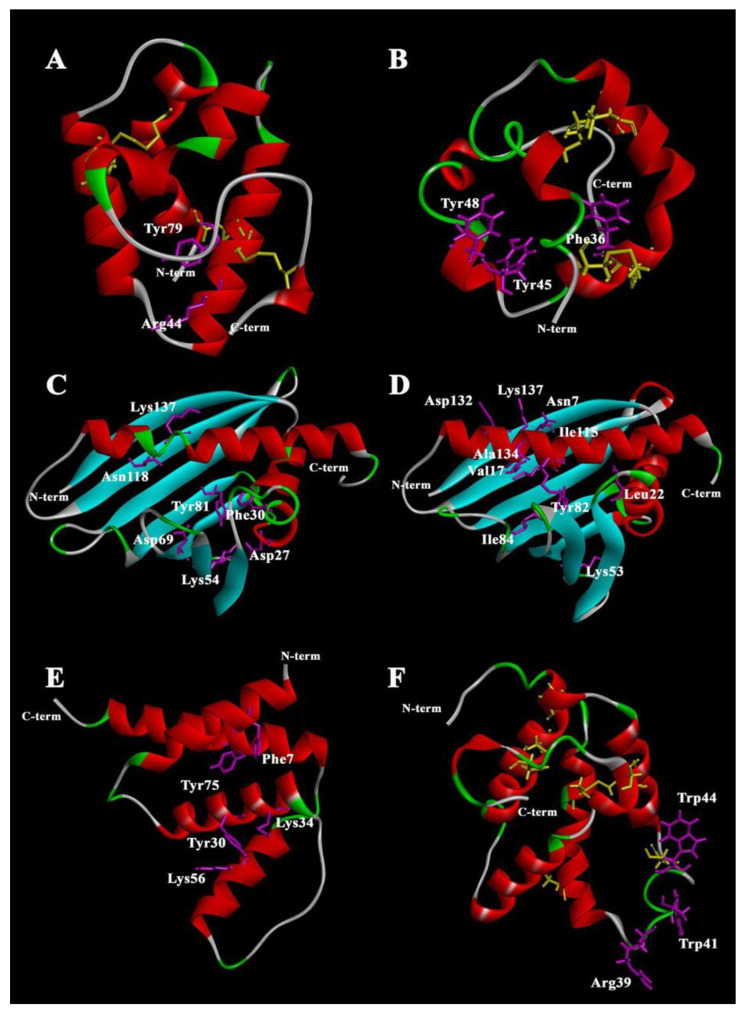
Spatial structures of plant lipid-binding proteins. (**A**) Rice LTP1 [PDB: 1RZL]; (**B**) rice LTP2 [PDB: 1L6H]; (**C**) birch Bet v 1 [PDB: 4A88]; (**D**) yellow lupin LlPR-10.1A [PDB: 4RYV]; (**E**) rice acyl-coa-binding protein 2 [PDB: 5H3I]; (**F**) PIN structure prediction using iterative threading assembly refinement I-TASSER. Key amino acid residues participating in ligand binding are highlighted in magenta. Cysteines and cysteine bridges are highlighted in yellow. The structures were visualized in Discovery Studio Visualizer (Dassault Systèmes BIOVIA, Discovery Studio Visualizer, v20.1.0.19295, San Diego: Dassault Systèmes, 2020).

Plant lipid-binding and transfer proteins of LTP, PR-10, ACBP, and PIN classes have a relatively low molecular weight (7–30 kDa) (Table 1). Amino acid sequences of the proteins of these four classes are not characterized by significant structural homology. At the same time, they all have similar spatial structure formed by α-helical and β-structural regions which is characterized by the presence of a hydrophobic cavity (Figure 1). Inside the cavity, the ligand-binding site is located.

These proteins are not characterized by ligand specificity, the reason for which may be the plasticity of the hydrophobic cavity. They reversibly bind a broad range of hydrophobic molecules of different chemical structures due to which they perform a variety of functions in plants. The proteins of these classes have different localizations and provide the necessary solubility of hydrophobic substances in the extracellular and intracellular spaces and enable movement of these substances throughout the plant.

LTPs, PR-10, ACBPs, and PINs interact with ligands according to the cooperative binding model, but the binding mechanisms have not yet been fully established. Positively charged and hydrophobic amino acids such as Tyr and Trp are most often key for ligand binding. As shown, their replacement leads to a structural rearrangement of these proteins and influences the specificity and ability to bind and transfer ligands.

Despite the similarities, plant lipid-binding and transfer proteins of these four classes have individual structural and functional features which are discussed in more detail below.

### 2.1. Lipid Transfer Proteins

LTPs were discovered about 40 years ago and they represent one of the most studied classes of plant proteins that bind and transport lipid molecules [9]. LTPs have been assigned to the class of plant stress proteins as PR-14 proteins (pathogenesis-related, PR) [10]. Although different classification systems have been proposed, LTPs can be divided in a simple way based on their molecular weight in two subfamilies: the 10 kDa (~90 to 95 amino acids) LTP1 group members and the 7 kDa (~70 amino acids) LTP2 group members [11]. In the LTPs, four conserved disulfide bridges, formed by an eight-Cys motif (8 cm) with the general form C-Xn-C-Xn-CC-Xn-CXC-Xn-C-Xn-C, stabilize the folding of three or four α-helices into a 3D structure (Figure 1A,B) [12]. The structural elements of the molecule form a large tunnel-shaped (for LTP1s) or conical-shaped (for LTP2s) internal cavity, which accommodates various types of lipids, and also exhibit unusual stability against thermal and digestive processing [13]. The cavity is highly flexible, allowing the protein to accommodate both single and double fatty acyl chain lipids [14]. After ligand binding, the volume of the protein cavity can increase two to four times [12,15]. The increase in cavity size is the result of conformational changes in the unstructured C-terminus of the protein. The LTP2 cavity has a pronounced flexibility compared to LTP1, which allows the placement of large ligands with a rigid structure, such as sterols [13]. The ability of LTPs to form complexes with ligands in vitro depends on the size of the hydrophobic cavity and the nature of the amino acid residues that form it, the structural organization of the ligand, and also on the experimental conditions (pH, buffer composition, temperature, etc.).

A number of amino acid residues located inside and at the entrance to the hydrophobic cavity play a key role in protein–lipid interactions [5]. In most LTP1 proteins, Arg44 (numeration according to LTP1 from *Oryza sativa* rice (PDB ID 1RZL)) residue is located near the entrance; this arginine interacts with polar head groups of lipids [16]. In LTP1 of rice, another basic residue, Lys35, is also involved in the interaction. For some LTP1s, Tyr79 residue can form hydrogen bonds with the polar head group of lipid ligands [17,18]. In the case of LTP2, the side chains of Phe36, Tyr45, Tyr48, and Tyr80 (numeration according to rice LTP2 (PDB ID 1L6H)) are the key ones in ligand binding [13].

Despite the similarity of the spatial structures of LTPs, these proteins are known to have different abilities to bind and transfer ligands. Some representatives of LTPs do not interact with lipids [19], probably due to the absence of a single cavity inside the protein molecule, while hydrophobic cavities of other LTPs can host two or three ligands at a time [20]. LTP1 has been found to interact with ligands according to the cooperative binding model, when the number of binding sites of protein that are occupied by a specific type of ligand is a non-linear function of this ligand’s concentration. The orientation of ligands simultaneously located in the hydrophobic cavity of the protein can vary [21,22].

In vitro binding experiments showed that LTPs can bind both saturated and unsaturated free fatty acids (C12–C20) or the fatty acyl chains presented in various molecules as in lysophosphatidylcholine (LPC), phosphatidylglycerol (PG), acyl coenzyme A (CoA), and cerebrosides (galactolipids), and it can bind jasmonic and abscisic acids, prostaglandin B2, molecules of organic solvents, and some drugs [18,21,23,24,25,26]. For many LTPs, in vitro binding data show that the proteins lack specificity in binding to ligands. They probably cannot properly reflect which ligands interact with LTP in the plant in vivo. However, for lentil Lc-LTP2, higher affinity for lipids with a negative charge and small hydrophobic chains was shown [26,27]. Recent studies on the *Juglans regia* walnut LTP1 have shown that this protein can bind exclusively oleic acid [28]. Arabidopsis LTP2 has a higher affinity for LPC derivatives with longer fatty acyl chains (C18) compared to shorter acyl chains (C14) [29]. One of the reasons for the specificity of ligand binding is the size of the hydrophobic cavity, which differs significantly in different representatives of the LTP class. At the same time, it is possible to change the specificity of ligand binding of LTPs using site-directed mutagenesis of the key amino acids [30,31]. 

To date, several LTP1 complexes with natural ligands have been isolated from plants. One of them is the covalent adduct of barley *Hordeum vulgare* LTP1 with an oxylipin, which is formed by the interaction of the carboxyl group of the Asp7 residue with allene oxide in the 9(*S*),10-epoxy-10,12(*Z*)-octadecadienoic acid molecule [32]. As a result of this reaction, α-ketol-9-hydroxy-10-oxo-12(*Z*)-octadecenoic acid is formed. For the allergen Pru p 3 from the peach *Prunus persica*, a non-covalent complex with 10-hydroxycamptothecin linked by an amide bond to the phytosphingosine tail has been described [33]. It has also been shown that LTPs from *Triticum aestivum, Artemisia vulgaris, Parietaria judaica,* and *Olea europaea* share a similar ligand when isolated from the natural source: a camptothecin-like polar head bound to a tail of phytosphingosine [34]. On the one hand, such ligands may indeed be the only compounds that bind LTPs under natural conditions. On the other hand, the similarity of the isolated LTP–ligand complexes may be due to a method of purification common to all of the above-mentioned proteins.

In addition to their ability to bind ligands, LTPs can also transfer lipid molecules between membranes in experiments in vitro. Particularly, they are able to transport phospholipids and their derivatives, as well as CoA derivatives [21,35,36]. It was shown that the ability of LTP2s to transfer lipids is several times higher than that of LTP1s [37]. Some LTPs damage cells of phytopathogens and model membranes [27,38], while there is no correlation between the ability of proteins to bind and transfer lipids and to influence the membrane permeability [39].

Despite a large number of studies on the mechanism of LTP lipid uptake and transport, it has not yet been definitively established. Ligand binding includes its uptake and retention inside the protein cavity. Initial protein–ligand interactions play the key role in the lipid uptake. To date, several variants of LTP interaction with ligands have been proposed. It was shown that myristic acid binds to the N-terminal part of LTP from *Solanum melongena*, leading to partial unlocking of the protein hydrophobic cavity and ligand internalization thereinto [40]. On the other hand, the lentil Lc-LTP2 most probably binds the surface of LPPG micelle by the positively charged “bottom” entrance of its hydrophobic cavity located near the C-terminal tail of the protein, and after that the lipid penetrates into the protein cavity [21]. Many studies argue that the presence of a polar head on a lipid favors the entry of aliphatic chains into the protein cavity. It has been shown that lipid ligands enter the internal cavity of LTP in a specific orientation [29,31]. At the same time, the specific site in the protein for initiating this interaction probably depends on the LTP [14].

The mechanisms of lipid transfer involving plant LTPs have not yet been elucidated. Supposedly, LTP binds the lipid molecule and then the protein–lipid complex interacts with the membrane, which results in lipid exchange [5,11].

Similar to the mechanism of interaction with lipids, the biological role of LTPs remains rather unclear. However, accumulating evidence shows that these proteins are essential for deposition and function of wax- and lipid-based polymers (suberin, sporopollenin) [41], seed development and germination [42,43,44], cell wall growth [45], defense signaling [46,47], and plant responses to biotic and abiotic stress [48,49,50].

### 2.2. Pathogenesis-Related Class 10 (PR-10) Proteins

To date, PR-10 class proteins are divided into two subclasses: intracellular pathogenesis-related proteins, or IPRs, exhibiting ribonuclease activity, and (S)-norcoclaurine synthases (NCSs). Proteins of the IPR subclass are also divided into several groups, including the group of homologs of the main birch pollen allergen, Bet v 1, homologs of major latex proteins, or MLPs, and cytokinin-specific binding proteins, or CSBPs, as well as recently discovered receptors of abscisic acid [51]. 

PR-10 proteins are 154 to 163 amino acids long and have a molecular weight of approximately 15–18 kDa. Their spatial structure consists of antiparallel, seven-stranded β-sheets wrapping around an amphipathic C-terminal α-helix (α3) 25 a.a. long embraced by two short α-helices (α1, α2) in the N-terminal region. A conserved P-loop motif (GxGGxGxxK) is present between β2 and β3. The main structural feature of the PR-10 fold is a large solvent-accessible hydrophobic internal Y-shaped cavity spanning the entire protein [52] (Figure 1C,D). The size of the cavity is disproportionately large for proteins of this size and the amino acids forming it are not strictly conserved [51]. The PR-10 proteins have inherent structural flexibility, which is likely due to the process of reorganization of the molecule required for ligand entry and/or release. However, the structural flexibility has been shown to be unevenly distributed along the protein backbone [53]. In accordance with the size of the hydrophobic cavity of PR-10, a variant of their classification was proposed. The type 1 cavity has members of the PR-10 class that specifically bind ligands and are involved in signaling. This cavity is characterized by a rather small size, a single entrance, and a single binding site. The type 2 cavity is typical of the PR-10 class that non-specifically binds various ligands and can act as storage proteins. The size of the cavity corresponds to the entire volume of the inner part of the protein, which makes it possible to accommodate two or more ligand molecules [54]. 

In experiments in vitro, the members of the PR-10 class have been shown to bind a broad range of hydrophobic to amphipathic ligands, differing in size and shape, to distinct binding sites within their hydrophobic cavity. These proteins are able to bind flavonoids, cytokinins, saturated and unsaturated fatty acids, brassinosteroids, sterols, and emodin [53,55,56]. Using crystal structures of Bet v 1a and Bet v 1j (Bet v 1.0801) with such ligands as 1-anilino-8-naphthalenesulfonate (ANS), deoxycholate, kinetin, and naringenin, it was shown that these compounds occupy different regions inside the hydrophobic cavity [57]. Due to the large conformational variability and the difference in the sequence of amino acid residues of the C-terminal helix, the cavities differ in shape and volume. Even between structurally similar isoforms of Bet v 1 homologs under the same experimental conditions, different binding abilities are manifested, which may be due to differences in the parameters of their hydrophobic cavities [58].

A number of amino acid residues in the Bet v 1 structure play an important role in the formation of a complex with ligands: Asp27 is presumably involved in the formation of hydrogen bonds with positively charged lipids [59]; Phe30 directly affects the topology of the hydrophobic region and is critical in various isoforms where it prevents the binding of brassinolide and related phytosteroids [57]; Lys54, Asp69, and Tyr81 are presumably the most important for kinetin binding [59]; for fatty acids and SDS, the key amino acids are Asn118 and Lys137 localized near the H2 tunnel [60], where Lys137 is involved in the formation of the second entrance to the molecule. Only two Bet v 1 homolog complexes with their natural ligands have been isolated from plant cells. A complex of Bet v 1 with flavonoid quercetin-3-O-soforoside (Q3OS) was isolated from birch pollen [60]. For the hazelnut allergen Cor a 1, the natural ligand is quercetin-3-O-(2“-O-β-D-glucopyranosyl)-β-D-galactopyranoside and highly similar to the birch Bet v 1 co-purified Q3OS, differing in the orientation of the hydroxyl group in the first sugar moiety. There is an interesting feature in Bet v 1 homolog interaction with its ligands. On the one hand, the Cor a 1 ligand does not interact with Bet v 1 and vice versa, despite the high structural and sequence similarity between both allergens. In addition, Q3OS binds only to the main isoform Bet v 1.0101 and not to other isoforms [61]. These observations are consistent with the idea that the presence of many PR-10 isoforms in the plant is due to their multifunctionality [62]. On the other hand, non-specific binding of flavonoids (e.g., quercetin, genistein, apigenin, daidzein, resveratrol) has been shown for Bet v 1 and Cor a 1 [6].

Of all the representatives of PR-10, only for homologs of Bet v 1 is there evidence about their possible involvement in the transfer of ceramides, sphingomyelins, and natural steroids. Bet v 1 was demonstrated to bind dioleoylphosphatidylcholine (DOPC) and dioleoylphosphatidylglycerol (DOPG) vesicles with different affinities depending on the pH. The question whether there is a difference in the manner of interaction of Bet v 1–ligand complex and free protein with the membrane remains open. Supposedly, free Bet v 1 reversibly binds membranes, but the complex formation can facilitate its penetration across the membrane [63].

The mechanism of binding and transfer of ligands for PR-10, as well as for LTPs, has not been fully studied. An attempt to describe the process of binding a PR-10 representative with a natural ligand was made for the LlPR-10.1A protein of *Lupinus luteus* with trans-zeatin, a natural plant phytohormone belonging to the class of cytokinins [64]. The hydrophobic cavity was found to accommodate three molecules of trans-zeatin in the crystal structure of LIPR-10.1A. The key amino acids of the protein participating in binding with trans-zeatin molecules are: Asn7 and Ile115 linked with the purine part and Leu22 and Tyr82 linked to the aliphatic part of the first molecule of trans-zeatin; Tyr82, Ile84, and Lys137 participating in binding with the purine part while Lys53 binds the aliphatic part of the second molecule of the ligand; finally, Val17 and Ala134 bind to the purine part while Asp132 binds hydroxyl of the third molecule of trans-zeatin. It is assumed that the binding process begins with the penetration of the two trans-zeatin molecules into the hydrophobic cavity through the main entrance. This process leads to a change in the structure of the protein and the opening of a second entrance providing space for a third molecule. The key to zeatin binding is the restructuring of the α3 helix in the protein. An unfolded helix of a ligand-free protein provides access to its hydrophobic cavity, and after binding to a ligand, the helix coils and blocks the exit of the ligand from the cavity [64].

For the PR-10 group members, their biological function in plants is due to their ability to bind certain ligands. These proteins can act as a kind of storage of regulatory and protective hydrophobic molecules and provide, if necessary, their rapid release. In addition, PR-10 proteins are involved in enzymatic processes [58,65], plant protection from biotic and abiotic stress [66,67,68], secondary metabolite biosynthesis [69], and storage and transport of small polar molecules [56,70].

### 2.3. Acyl-CoA-Binding Proteins

Acyl-CoA-binding proteins (ACBPs) represent a common class of proteins involved in acyl-CoA metabolism in most eukaryotic and prokaryotic organisms. 

Since the identification and systematization of ACBPs are currently complicated due to the lack of a unified nomenclature of these proteins, to date, plant ACBPs have been classified according to their molecular weight and domain architecture [71,72]. Plant ACBPs can be divided into four categories. Class I (small ACBPs) includes proteins that consist of 88–155 amino acids and contain only one ACB domain. Class II (ankyrin-ACBPs) is usually composed of 260–370 amino acids and additionally contains ankyrin repeats at the C-terminus of the protein. Class III (large-ACBPs) comprises large proteins with a single C-terminal ACB domain, which is usually composed of 215–700 amino acids. Class IV (kelch-ACBPs) involves multidomain proteins which typically consist of 648 to 668 amino acids and contain C-terminal kelch motifs [63]. 

The ACB domain is the most structurally conserved part of the ACBP and common to all ACBPs [73]. The ACB domain structure consists of four α-helices in an up–down–down–up arrangement, in which helices 1, 2, and 4 (numbered from the N-terminus) are more closely packed together and helix 3 is in close contact with helix 2 only [74].

This structural organization of the molecule is conserved in all ACBPs of yeast [75], *Plasmodium falciparum* [76], armadillos [77], humans [78], and rice [79]. An exception is *Moniliophthora perniciosa* ACBP, which contains an additional fifth helix at the C-terminus and helices 1, 2, 4, and 5 or helices 1–4 can form a classic four-helix bundle [80]. To date, spatial structures have been established only for two structurally similar isoforms of an ACBP from rice (OsACBP1 and OsACBP2) (Figure 1E). They have a deep and narrow groove, which is largely hydrophobic in nature. This groove contains the acyl-CoA ester-binding site. The ligand binding site consists of three regions dedicated to the acyl moiety of the ligand, the adenine ring, and the 3’-phosphate of the ligand, respectively [79]. Despite the high similarity of rice OsACBP1 and OsACBP2 sequences, their ability to bind saturated and unsaturated acyl-CoA esters (C16–C18) differs significantly [79]. As with other lipid-binding proteins, the variability in the binding of ligands to ACBPs is determined by the amino acids that form the binding site. Upon alignment of the acyl-CoA-binding domains of ACBPs from plants, yeasts, and animals, 19 amino acid residues were proposed to be constitutive in all species considered. Potential acyl-CoA ester-binding sites imply five amino acid residues that correspond to Phe 7, Tyr 30, Lys 34, Lys 56, and Tyr 75 (numbering for OsACBP2) [81]. The YKQA and KWDAW motifs required for acyl-CoA ester binding were conserved across all species [82].

In vitro binding experiments show that plant ACBPs can bind long- and very-long-chain saturated and unsaturated acyl-CoA esters (C14–C26) but with varying affinities. The strongest complexes are formed with acyl-CoA esters with a chain length of C18 and C20. In contrast to animal and yeast proteins, plant ACBPs, in addition to acyl-CoA esters, appear to be able to associate with at least one class of phospholipids such as lysoPC, DIPC, phosphatidic acid (PA), and phosphatidylethanolamine (PE) with saturated and unsaturated acyl tails with a chain length from C16 to C18 [71,83,84]. In addition to phospholipid binding, arabidopsis ACBPs (AtACBPs) were shown to interact with proteins. AtACBPs interact with various transcription factors that activate gene expression for abscisic acid (ABA) or ethylene responses upon perception of stress stimuli [85,86,87]. Moreover, for some plant ACBPs affinity for heavy metals was shown [88].

The ligand-binding modes among all ACBPs are diverse; for example, fungus *Moniliophthora perniciosa* and human liver ACBPs undergo dimerization [78,80], while bovine ACBP remains monomeric. For rice, OsACBP2 has been shown to bind C18:3-CoA as a monomer [89].

The proteins bind to acyl-CoA according to the cooperative binding model [90,91]. The probability of formation of the acyl-CoA–protein complex is largely dependent on the length of the acyl chain and the number of double bonds of ester [92].

Binding of lipids by ACBPs and their protein–protein interactions determine their functional activity in plants. These interactions are important for the regulation of abiotic and biotic stress responses [93,94,95], as well as plant development including embryogenesis [96], seed dormancy and germination [97,98,99], cuticle development [100], pollen growth [101], and senescence [102].

### 2.4. Puroindolines

Puroindolines (PINs) were first reported in 1990. PINs are a family of small proteins from wheat and barley which have attracted significant attention due to their role in determining the endosperm texture. PINs are 115 to 120 amino acids long and have a molecular weight of approximately 13 kDa. Two isoforms named puroindoline-a (PIN-a) and puroindoline-b (PIN-b) have been purified and characterized from wheat (*Triticum aestivum*). They exhibit more than 60% homology in their amino acid sequence [103,104]. Both PIN-a and PIN-b contain a backbone of 10 Cys residues including a Cys–Cys pair and a Cys–X–Cys triplet, and form a tertiary structure very similar to that of LTPs, comprising four α-helices separated by loops and stabilized by five disulfide bridges (instead of four, due to the two extra Cys compared to LTPs) (Figure 1F). Both proteins also contain a conserved Tyr residue in helix 1 which may be functionally important [105]. Both PIN-a and PIN-b contain a unique tryptophan-rich domain (TRD). PIN-a has a high content of hydrophobic Trp residues on its TRD and PIN-b has a high content of Leu on the outer surface of its two a-helices. For PIN-a, the sequence consists of eight amino acid residues: WRWWKWWK. A similar domain for PIN-b has a shorter amino acid sequence: WPTKWWK [104]. The Trps occupy a surface loop, the TRD forming an extension of it, stabilized by the Cys28–Cys48 disulfide bridge in PIN-a and the Cys29–Cys48 one in PIN-b [105]. Trps in both isoforms, PIN-a and PIN-b, are accessible to the solvent. Trp is hydrophobic and able to form hydrogen bonds with polar components; that is why it is often located at the hydrophilic/hydrophobic interface. According to the number of Trps, the loop is longer and more flexible in PIN-a [106]. To date, the high-resolution structure of PINs remains to be solved, mainly due to problems with their crystallization and difficulties in obtaining a stable non-aggregated solution required for NMR structural characterization.

Both PIN-a and PIN-b have the ability to interact with both lipid monomers and aggregates (micelles or liposomes) of unique wheat phospholipids and glycolipids [107,108]. Presumably, the proteins have three lipid-binding regions [109]. This probably indicates that the interaction with lipids in puroindolines does not occur due to the immersion of the lipid molecule inside the hydrophobic cavity, as in the case of LTPs and other proteins described in this review. Moreover, the number of lipid-binding regions is different from the actual number of lipid molecules that can be bound. It is assumed that TRD, in which the key residues are Trp and Arg, is responsible for the binding of lipid molecules in the PIN structure [110]. In PIN-a, only Trp41 and Trp44 appear to be crucial for its binding to the yeast plasma membrane. In PIN-b, none of the three Trps play a critical role in membrane interaction [111]. However, the lower content of Trp in the structure of PIN-b causes a higher affinity of the proteins of this group for phospho- and glycolipids of the endosperm compared to PIN-a. As in the case of LTPs, point mutations in the structure of PIN-b affect its ability to bind lipids. For example, wild-type PIN-b has been shown to have higher selectivity for 1,2--dipalmitoyl-sn-glycero-3-phosphoglycerol (DPPG) than mutant analogs PINB G46S and PINB W44R [112].

The complexes of PINs with polar lipids are stabilized by ionic, hydrogen, and hydrophobic interactions. For PIN-a, interactions with model phospholipid bilayers and micelles were shown to depend on the head group, acyl chain length, ionic environment, and lipid-to-protein ratio [113]. In contrast to LTPs, to interact with lipids, PINs require the presence of a phase interface. For example, the interaction of PINs with lysoPC occurs only when the critical micelle concentration is exceeded [114]. Moreover, PIN-a has been suggested to form cation selective ion channels in biological membranes in a voltage-dependent manner [115,116].

The biological function of puroindolines is not completely clear but in plants their function is probably determined by their interaction with membranes. It has been shown that these proteins play an important role in protecting plants from pathogens [117,118]. 

## 3. Possible Applications of Plant Lipid-Binding Proteins

### 3.1. Protein-Based Drug Delivery System

Lipid transfer proteins as well as human lipid-binding proteins calycins [119] are considered as possible drug delivery systems of unstable and water-insoluble drugs due to the following reasons. These proteins are characterized by high stability and bind a wide range of hydrophobic molecules, including drugs of various pharmacological groups. Ligand-binding affinity and specificity of LTPs can be improved by modifying their structure. Biologically active molecules placed inside the hydrophobic cavity of these proteins can be protected from oxidation or degradation and released slowly from the ligand–protein complexes. Using virtual screening and molecular docking analysis, maize LTP1 has been shown to be able to accommodate drugs with a phenyl head group and a tail of eight carbons in its hydrophobic cavity [120]. LTP2 of rice is capable of high-affinity binding with such antiviral purine analogs as acyclovir and vidarabine [121]. Using fluorescence binding assay, it was shown that wheat LTP1 can form complexes with various ligands for cosmetic or pharmaceutical applications, i.e., skin lipids such as sphingosine, sphingomyelin, and cerebroside; azole derivative BD56 having antitumoral and/or antileishmanial properties; or antifungal drug amphotericin B [24]. At the same time, rice LTP2, having a more voluminous hydrophobic cavity, can bind to such sterol-like molecules as a cholesterol-lowering agent β-sitosterol [120].

### 3.2. Food Industry

Barley LTPs, LTP1 and LTP1b, are the key factors in the brewing process due to their participation in fermentation and beer foam stabilization. Barley LTP1 retains its structure and ability to interact with lipids upon heating during beer pasteurization, whereby it reduces the negative impact of lipids on the formation and stability of foam [122]. The surfactant properties of LTP1 are increased upon brewing due to protein glycosylation and acylation. On the other hand, as shown, LTP1 exhibits antiyeast activity and can negatively influence the fermentation process [123]. LTP1b is a lipid-bound isoform of LTP1 (LTP1-9(*S*),10-epoxy-10,12(*Z*)-octadecadienoic acid), which is formed during fermentation and does not inhibit yeast growth due to the presence of fatty acid in their structure [124]. Thus, the optimal balance between two LTP isoforms’ content is a necessary condition for obtaining high-quality beer [125].

It is well known that kernel hardness mainly determines end-use qualities of wheat cultivars. As shown, wheat puroindolines PIN-a and PIN-b play an important role in the grain texture and are markers available to improve wheat hardness in breeding programs [126]. The expression of both *Pina* and *Pinb* genes, encoding puroindolines PIN-a and PIN-b, is necessary for the soft-kernel phenotype, whereas deletion or loss-of-function mutations in any of these genes lead to hard endosperms [127]. The presence of starch-bound PIN-a and PIN-b (friabilin) rather than total PINs’ content affects the hardness of wheat grains [128]. It was supposed that wheat puroindolines bind polar lipids on the surface of starch granules, preventing adhesion between the starch grains and the protein matrix [128,129]. Recently, it was demonstrated that protein–protein interactions can also play an important role in wheat grain texture. The interaction of PIN-a with a monomeric gliadin (prolamin) induces gliadin aggregation and prevents further interaction of the storage prolamins with starch granules [129].

### 3.3. Plant Stress Tolerance

As mentioned above, lipid-binding proteins of all classes described play an important role in growth and development of plants, as well as in their protection under abiotic and biotic stress conditions. Thus, the use of various strategies ensuring a high level of expression of these proteins in plant tissues can significantly increase the resistance of crops to infections and such adverse factors as drought and salinity, and, as a result, minimize crop losses.

Puroindolines are amphipathic proteins possessing antibacterial and antifungal properties and protect crops from different pathogens due to the presence of characteristic tryptophan-rich domains (TRDs) in their structure [130]. Synthetic peptides based on the TRD of wheat PIN-a and PIN-b realize their antimicrobial effects through pore formation in the cell membrane, followed by intracellular mechanisms of activity [131]. Transgenic plants, carrying puroindoline genes, exhibit increased resistance to fungal pathogens throughout the plant rather than it being limited to seeds [128]. Mold causes not only plant infections, but also reduces the quality of stored grains due to production of toxins that pose a potential threat to human health. PINs effectively inhibit the growth of molds (various species of *Penicillium, Aspergillus, Alternaria*, and *Fusarium* genera) as well as toxin accumulation in stored grains, such as wheat and rice. Therefore, these proteins are considered as eco-friendly antifungal agents which may ensure safe and high-quality foods during storage [132].

Plant acyl-CoA-binding proteins (ACBPs) bind phospholipids and acyl-CoA esters and play an important role in lipid metabolism due to which these proteins are involved in plant adaptation to such stresses as drought, adverse temperatures, salinity, oxidation, hypoxia, heavy metals, wounding, and pathogens [83]. Transgenic plants overexpressing different ACBPs are characterized by abiotic and biotic stress tolerance [133,134]. Protective effects of ACBPs are associated with their participation in different signaling pathways including responses mediated by ethylene, abscisic, salicylic, and jasmonic acids [83]. It has been suggested that under drought and heavy metal stresses, ACBPs take part in cuticle formation [100] and phytoremediation [135], respectively. Upon infection, these proteins may activate synthesis of different classes of pathogenesis-related proteins (PRs), H_2_O_2_ production, and cell death [136]. In addition, ACBPs are likely to be phloem-mobile proteins that affect the pool of fatty acids and jasmonate content in the phloem [93].

Both lipid transfer proteins (PR-14) and PR-10 belong to the large family of pathogenesis-related proteins, the synthesis of which is activated in plants under various stress conditions. These proteins perform various functions, but the most important of them is plant adaptation and survival under abiotic and biotic stress. Mainly extracellular localization is typical of LTPs, whereas PR-10 typically functions inside cells [137]. These proteins not only are involved in intracellular and apoplastic lipid transport, but also, similar to ACBPs, act as systemic transporters of hydrophobic compounds via phloem and xylem vessels [138,139]. Both classes of proteins, in addition to the ability to bind and transfer hydrophobic ligands, are characterized by a number of biological activities [137]. Some members of the LTPs and PR-10 classes display antifungal and antibacterial activity, increasing the permeability of the cell membranes of pathogens [38] or showing RNase activity [140], respectively. Both LTPs and PR-10 are involved in the defense signaling in plants under infection [141,142]. Transgenic plants overexpressing LTPs or PR-10 are often characterized by enhanced tolerance to various stresses including fungal infections [143,144], salinity [143,145], and drought [146].

### 3.4. Diagnosis and Treatment of Allergic Disease

Some representatives of PR-10, namely homologs of Bet v1, as well as LTPs, are known as food or pollen allergens. Homologs of Bet v 1 typically cause a local allergic reaction, while LTPs are causative agents of not only local, but also systemic, allergic reactions [137]. To date, allergens of these classes are used for a comprehensive analysis of sensitization and determination of cross-reactivity profiles of patients with food and pollen allergy.

In addition, allergens of homologs of Bet v1 and LTPs are investigated as a possible alternative to plant extracts for allergen-specific immunotherapy (ASIT). The use of hypoallergenic variants of plant allergens is considered the most promising approach to ASIT to reduce the risk of IgE-mediated side effects [147]. Hypoallergenic analogs of Bet v 1-related allergens (Gly m 4 [148], Pru av 1 [149], Mal d 1 [150]) were engineered by directed mutagenesis and produced. rBet v 1-FV became the first hypoallergenic variant of PR-10 family allergens, and the first trial on humans was started [147]. Several hypoallergenic LTPs (e.g., Par j 1/Par j 2 [151] and Pru p 3 [152]) were developed for more safe immunotherapy as well.

Not only site-directed mutagenesis, but also interaction with ligands can presumably change the immunogenicity of Bet v 1 and LTP homologs, since proteins undergo structural rearrangements upon binding to ligands [21,64]. For allergens of LTP class Tri a 14 from *Triticum aestivum* and Pru p 3 from peach, lipid binding increased their sensitivity to proteolytic enzymes of the gastrointestinal tract [153] and probably decreased immunoreactivity of these food allergens.

## 4. Conclusions

We reviewed four classes of the best studied plant lipid-binding and transfer proteins, LTPs, PR-10, ACBPs, and PINs. These proteins carry out intracellular, extracellular, and systemic transport of lipids and other biologically active hydrophobic molecules due to which they perform a variety of functions in plants. Even within one class, there are several groups of proteins with different structural and functional features. However, all of them are united by a similar folded structure with an internal cavity having a special ligand-binding site. These proteins reversibly bind different lipid ligands without any pronounced specificity according to the cooperative binding model, but the binding mechanisms have not yet been fully established. At the same time, some key amino acids defining their binding capacity are present in the structure of all four protein classes. Their replacement leads to a structural rearrangement of these proteins and change in binding efficiency and spectrum of suitable ligands. This opens up new prospects for applications of plant lipid-binding proteins in medicine, cosmetology, the food industry, and agriculture, since their possible ligands can be substances with different biological activities.

## Figures and Tables

**Table 1:** Comparison of structure–functional properties of plant lipid-binding and transfer proteins.

Characteristics	LTPs	PR-10	ACBPs	PINs
Protein MW (kDa)	6–7 (LTP2s) or 9–10 (LTP1s)	15–18	9–70	13
Disulfide bonds	four conserved disulfide bridges	no	no	five conserved disulfide bridges
Spatial structure	three or four α-helices and a flexible C-terminal coil	three α-helices and seven antiparallel β-strands	four α-helices	four α-helices
Conservative motif	C...C...CC...CXC..C...C	glycine-rich loop or GXGGXGXXK motif (aa 47–55)	Acyl-CoA-binding domain, ankyrin repeats, or C-terminal kelch motif	tryptophan-rich domain
Volume of cavity	180–1000 Å3	2100–3900 Å3	550–800 Å3	900–1000 Å3 (according to cavity volume assessment with CASTp) *
Amino acid residues interacting with a ligand	Arg44 and Tyr79 (numeration for rice LTP1) Phe36, Tyr45, and Tyr48 (numeration for rice LTP2)	Asp27, Phe30, Lys54, Asp69, Tyr81, Asn118, Lys137 (numeration for Bet v 1)	Phe7, Tyr30, Lys34, Lys56, and Tyr75 (numeration for AtACBP6)	Arg39, Trp41, and Trp44 (numeration for PIN-a)
Ligand name	saturated and unsaturated fatty acids (C12-C20), PC, PG, acyl-CoA, cerebrosides (galactolipids), prostaglandin B2, molecules of organic solvents, some drugs	saturated and unsaturated fatty acids, flavonoids, cytokinins, brassinosteroids, sterols, and emodin	acyl-CoA, LPC, DIPC, PA, and PE with saturated and unsaturated acyl tails (C16-C26)	phospholipids and glycolipids
Localization	generally extracellular	generally intracellular and cytosolic	subcellular	subcellular

* Computed Atlas of Surface Topography of proteins (CASTp) is an online resource for locating, delineating, and measuring concave surface regions on three-dimensional structures of proteins (http://cast.engr.uic.edu (accessed on 15 November 2022).

## Data Availability

Not applicable.
